# Robotic-Assisted Kinematically Aligned Total Knee Arthroplasty

**DOI:** 10.7759/cureus.96170

**Published:** 2025-11-05

**Authors:** Alex M Hollenberg, Joseph T Gibian, William A Zuke, Adam M Holzmeister, Nicholas Brown, Andrew M Schneider

**Affiliations:** 1 Orthopedic Surgery, Washington University School of Medicine, St. Louis, USA; 2 Orthopedic Surgery, Western Michigan University Homer Stryker M.D. School of Medicine, Kalamazoo, USA; 3 Orthopedic Surgery, BoulderCentre for Orthopedics and Spine, Boulder, USA; 4 Orthopaedic Surgery & Rehabilitation, Loyola University Medical Center, Maywood, USA

**Keywords:** cartilage mapping, kinematic alignment, mechanical alignment, primary total knee arthroplasty, robotic knee surgery

## Abstract

The optimal alignment strategy in total knee arthroplasty (TKA) has yet to be elucidated. Mechanical alignment (MA), traditionally thought of as the gold-standard approach, positions implants perpendicular to the mechanical axes of the femur and tibia to achieve neutral limb alignment in the coronal plane. By contrast, kinematic alignment (KA) seeks to recreate the patient’s pre-arthritic joint line, limb geometry, and soft-tissue balance by resurfacing the distal femur and proximal tibia. Although multiple studies have reported either equivocal or improved functional outcomes for KA compared to MA, most KA techniques have relied on cartilage thickness assumptions and caliper-guided instrumentation, which can be limited by the precision of manual bone resections and may necessitate intraoperative bony recuts when residual soft tissue imbalance persists. To address these limitations, robotic-assisted TKA offers enhanced accuracy and reproducibility of implant placement by facilitating patient-specific bony resection planning, objective assessment of soft tissue balance, and intraoperative mapping of intact cartilage. In this article, we present our surgical technique for robotic-assisted kinematically aligned TKA, which leverages a CT-based robotic platform to map cartilage intraoperatively and guide patient-specific component placement in accordance with traditional KA principles.

## Introduction

Total knee arthroplasty (TKA) relieves pain and restores function in patients with end-stage arthritis by replacing diseased joint surfaces and restoring stable knee mechanics. Historically, implant alignment has been viewed as critical to achieving durable outcomes, as malalignment can lead to uneven load distribution, abnormal wear patterns, instability, and higher revision rates [1,2]. The traditional mechanical alignment (MA) philosophy - long regarded as the gold standard - positions the femoral and tibial components perpendicular to their respective mechanical axes to achieve neutral limb alignment in the coronal plane [1,3,4]. This approach was originally adopted not only to prevent catastrophic failure of early implant designs by evenly distributing load across the joint but also because early instrumentation and limited implant size options made uniform MA the most practical and reproducible goal. While MA has demonstrated excellent long-term implant survivorship [5], a subset of patients report dissatisfaction despite a technically successful surgery [6]. Enabling technologies such as computer navigation and robotics have improved the accuracy of achieving neutral alignment; however, this has not consistently translated into improved functional outcomes or satisfaction [7-10]. Meanwhile, with modern implant designs and fixation techniques, recent data suggest that deviation from neutral alignment may actually improve clinical outcomes without compromising implant longevity [11-14].

The pursuit for improved patient satisfaction after TKA has prompted a reevaluation of what constitutes “normal” alignment. Radiographic studies have demonstrated that many individuals have constitutional varus alignment [15] and that the native flexion-extension axis and joint line orientation do not reliably correspond to traditional mechanical reference axes [16]. These findings underpin kinematic alignment (KA) philosophy, which seeks to restore the patient’s pre-arthritic joint line, limb geometry, and soft-tissue tension rather than imposing a uniform neutral mechanical axis [4,15,17,18]. Proponents of KA suggest that this patient-specific strategy may reduce dissatisfaction by better restoring native knee kinematics [13,19]. Multiple studies have reported either equivocal or improved outcomes for KA relative to MA, although long-term survivorship remains under investigation [18-25].

Most existing KA techniques rely on a cartilage thickness assumption of 2 mm and caliper-guided instrumentation, which can be limited by the precision of manual bone resections and may require recuts if soft tissue imbalance persists [26]. Small inaccuracies can propagate, leading to unintended limb alignment, joint line position, or soft-tissue tension. Robotic-assisted TKA has emerged as a potential solution to this challenge, offering enhanced accuracy and reproducibility by enabling patient-specific alignment planning, intraoperative cartilage mapping to guide component position, and objective quantification of ligament tension, without reliance on jigs and instruments specifically designed for MA [27-29]. These capabilities make robotics well-suited for executing a KA strategy with consistency and precision.

The purpose of this technical report is to describe our surgical workflow for robotic-assisted kinematically aligned TKA, leveraging CT-based planning and intraoperative robotic guidance to restore native joint anatomy and soft tissue balance while reducing reliance on manual resection techniques or assumptions of cartilage wear.

## Technical report

Preoperative planning

**Figure 1:**
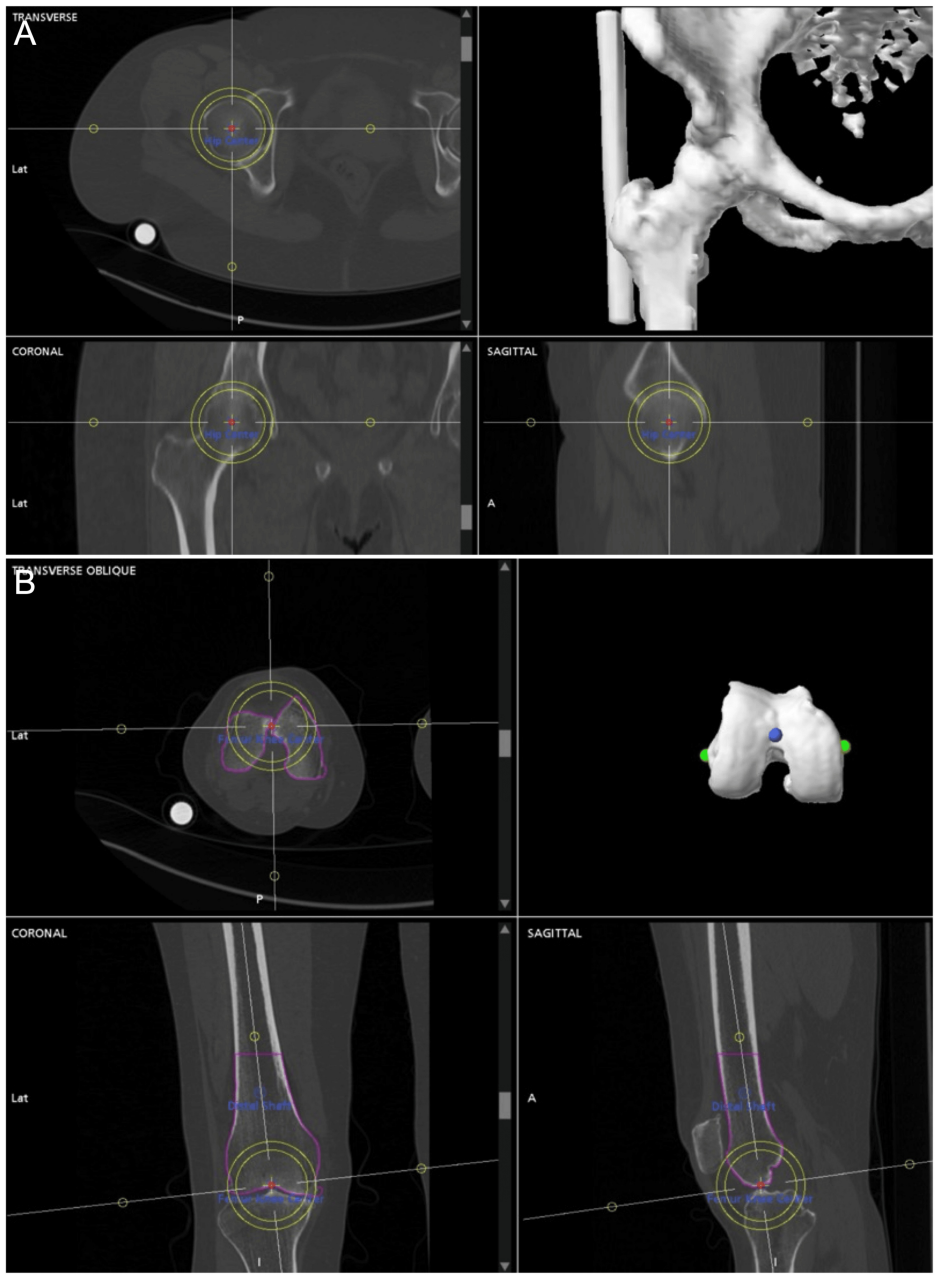
Defining the mechanical axis The mechanical axis is defined by a line between (A) the center of the femoral head and (B) the center of the knee. The center of the femoral head and the knee are both chosen in all three planes.

**Figure 2:**
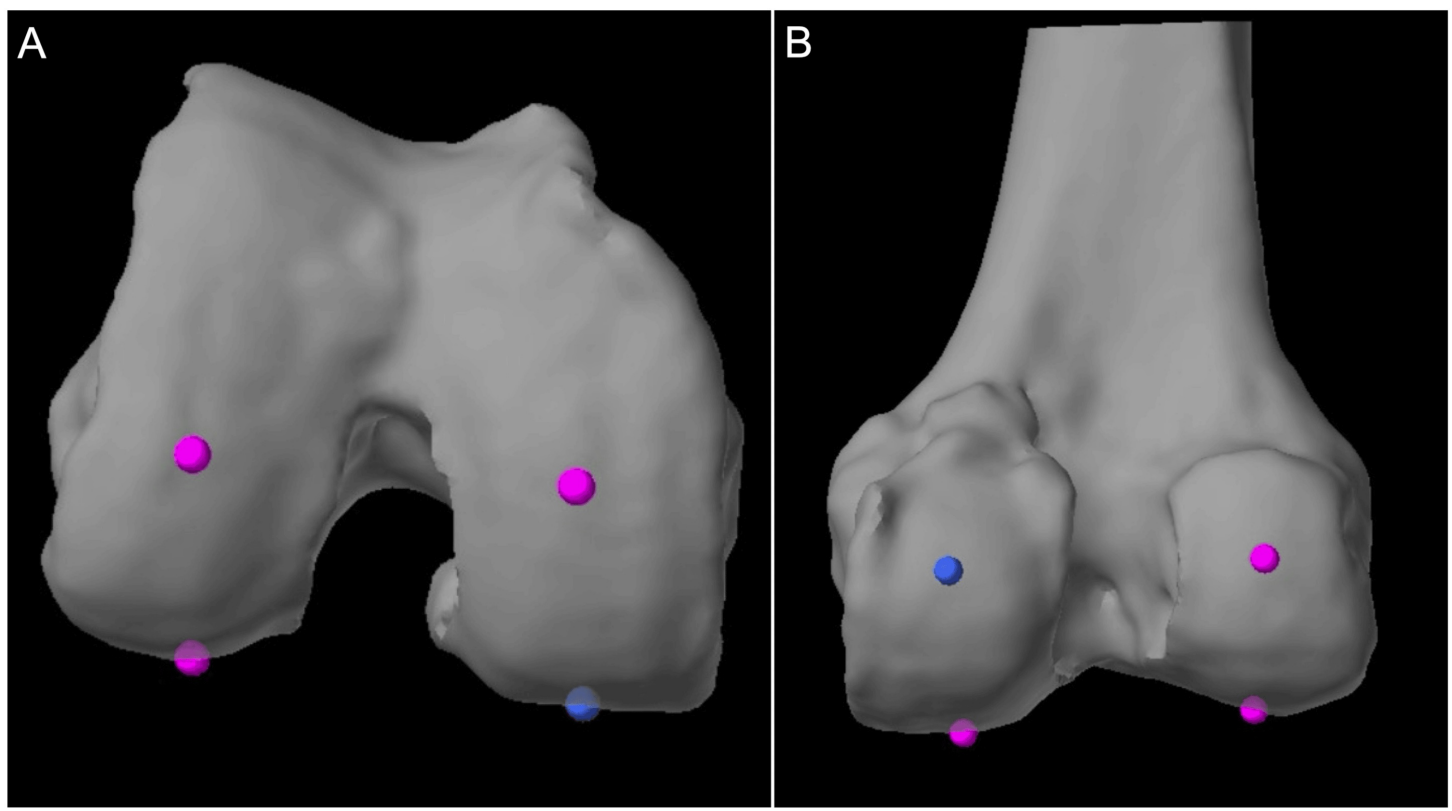
Correct distal femoral resection landmarks (A) The distal resection landmarks (magenta) are chosen based on the distal most aspect of the medial and lateral femoral condyles, to best represent native coronal plane joint line obliquity. (B) The posterior resection landmarks (blue and magenta) are chosen based on the posterior most aspect of the medial and lateral femoral condyles, to best represent the posterior condylar axis.

**Figure 3:**
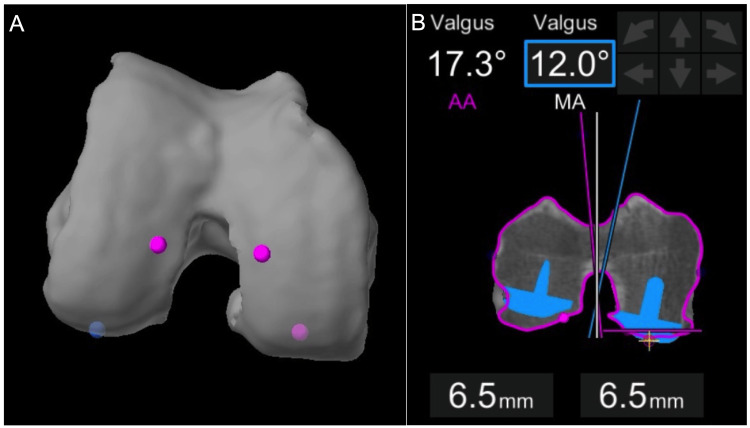
Incorrect distal femoral resection landmarks (A) These points on the distal femur (magenta) are too close to the notch and do not represent the true joint line obliquity of the distal femur. (B) Incorrectly chosen distal resection landmarks introduce false valgus into the distal femoral cut.

All patients undergo a preoperative CT scan, which is used to generate a three-dimensional model of their anatomy. Accurate selection of limb axes and resection landmarks is critical, as these points set the foundation for subsequent cuts that determine joint line obliquity and limb alignment. The mechanical axis is defined by selecting a point in the center of the femoral head and knee (Figure 1A, 1B). Distal resection landmarks are chosen at the most distal aspect of the medial and lateral femoral condyles, and posterior resection landmarks are selected at the most posterior aspect of the medial and lateral femoral condyles (Figure 2A, 2B). Inaccurate selection of these points introduces error, resulting in failure to reproduce native knee alignment (Figure 3A, 3B). The knee is then virtually sized, and components are digitally overlaid onto the patient’s knee. Using a KA strategy, our objective is to replace the pre-arthritic bone and cartilage with an equivalent amount of metal. As a preoperative starting point, we plan for 6.5 mm bony resections (including saw blade kerf) on the femur-medially and laterally, distally, and posteriorly. This assumes 2 mm of cartilage thickness for a sum of 8.5 mm, which is the thickness of the single-radius femoral implant used by the senior author. The actual distal and posterior cartilage thickness is quantified intraoperatively, and osseous resection depths are adjusted as needed before bone cuts are made. The tibial cut is provisionally set to best match the patient’s native anatomy in the coronal and sagittal planes.

Approach

With the knee flexed to 90 degrees, the skin is incised, the subcutaneous tissue is dissected, and a medial parapatellar arthrotomy is made. A release of the deep medial collateral ligament is performed, and the fat pad is resected. The patella is then inspected and selectively resurfaced. The anterior cruciate ligament is removed, and the posterior cruciate ligament is retained.

Pin and array placement

Four fingerbreadths distal to the tibial tubercle, two small stab incisions are made extra-incisional on the anteromedial surface of the tibia. The 3.2 mm self-drilling pins are drilled perpendicular to the tibia in a bicortical fashion, and the tibial array is secured. The 4.0 mm femoral pins are drilled intra-incisional unicortically approximately 1-2 cm proximal and 1-2 cm anterior to the medial epicondyle. The femoral array is attached to the pins and secured with a screwdriver.

Registration

The hip center is found by taking the hip through a circular range of motion. The ankle center is found by registering the medial and lateral malleoli. The femur is registered by placing a sharp probe directly on the bone at predefined landmarks displayed on the robotic screen. The accuracy of the femoral registration is then checked and verified. Tibial registration is carried out in a similar manner.

**Figure 4:**
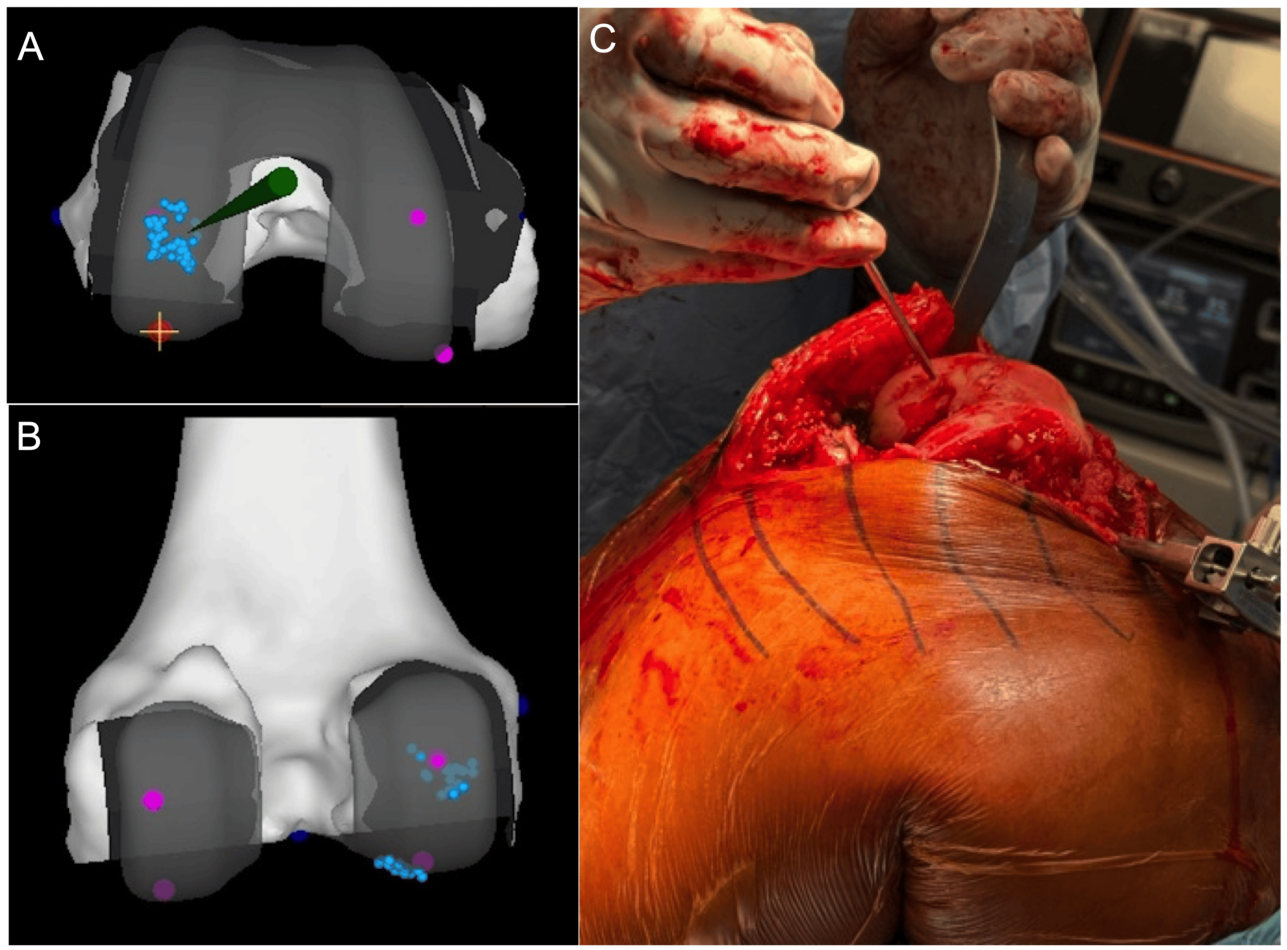
Intraoperative cartilage mapping The blunt probe (green) is used to map the surface of the unworn (A) distal and (B) posterior femur (blue dots). (C) Intraoperative photo demonstrating use of the blunt probe to map the unworn distal lateral femur.

The knee is stressed medially and laterally in both flexion and extension, with the resulting four gaps recorded by the robotic software. The unworn compartment of the knee is then inspected for the absence of cartilage wear. This step is critical for reproducing the native joint line, accounting for patient-specific cartilage thickness. Using a blunt probe, the intact cartilage on the unworn side of the femur is mapped by capturing a series of points along its distal and posterior surfaces, taking care to avoid any areas of damaged cartilage (Figure 4A-4C). This creates an arc of cartilage to be used as a template for where to position the femoral component. If no intact cartilage remains on either side, this step is omitted, and a pre-arthritic cartilage thickness of 2 mm is assumed. Once intact cartilage points are captured both distally and posteriorly on the unworn side of the femur, registration is complete.

Planning the cuts

**Figure 5:**
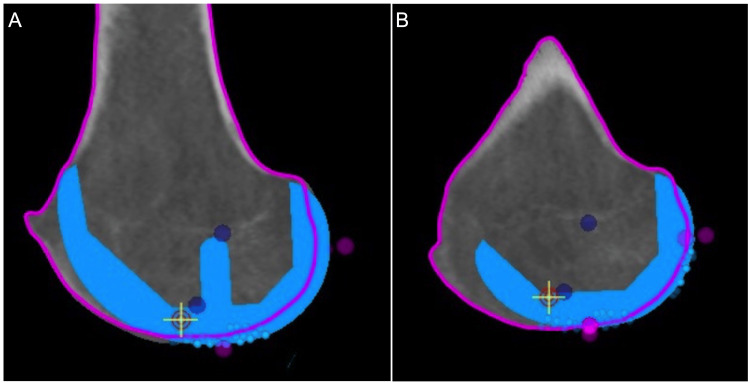
Aligning the femoral component The femoral component is aligned colinear with the mapped cartilage points (blue dots) both (A) distally and (B) posteriorly.

**Figure 6:**
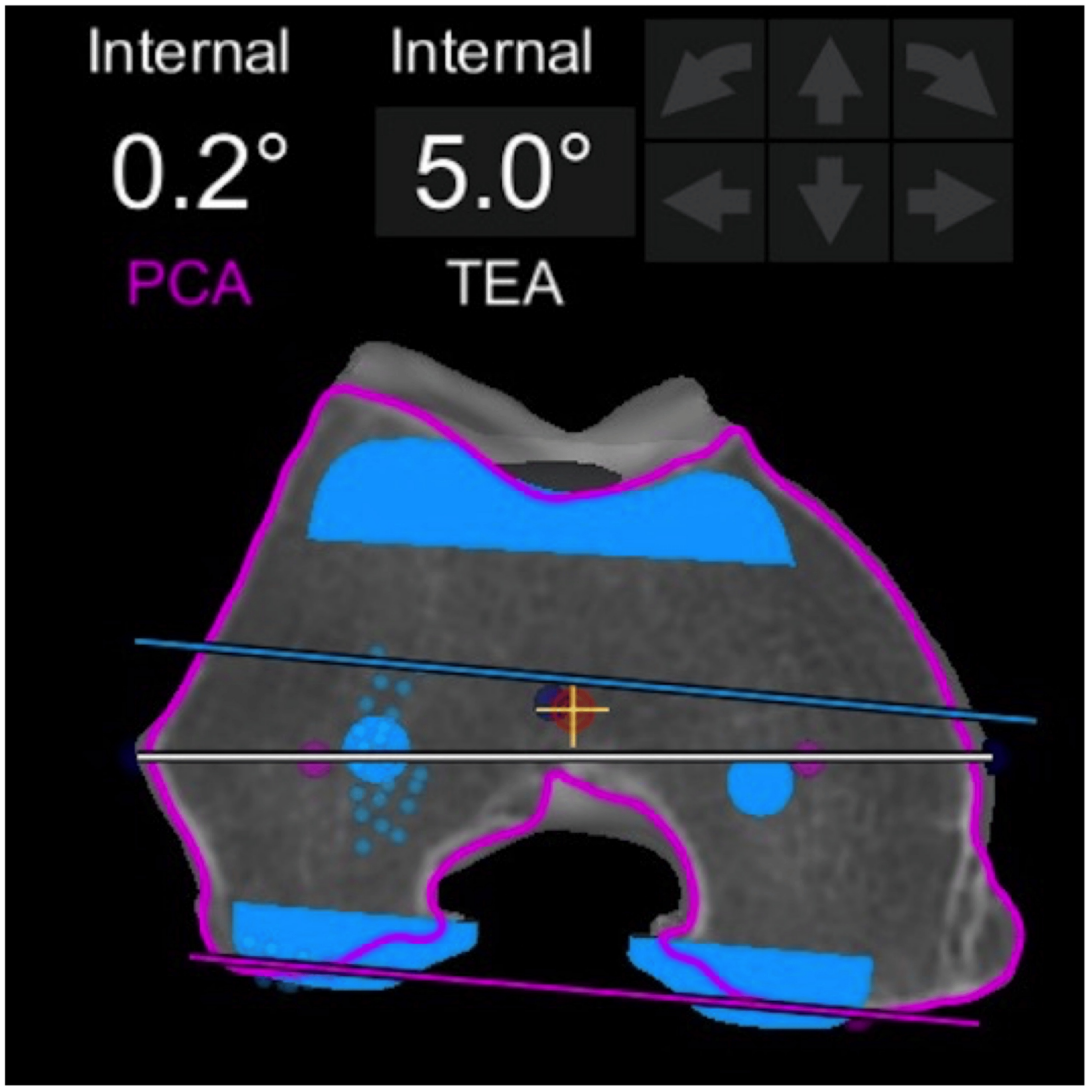
Femoral component rotation The rotation of the femoral component is parallel with the posterior condylar axis and matches with the trochlear position of the native femur, despite being 5 degrees internally rotated relative to the transepicondylar axis (TEA), an irrelevant landmark in KA philosophy.

The femoral component on the unworn side of the knee is viewed in the sagittal plane and adjusted on the robotic software to align with the previously mapped cartilage points, both distally and posteriorly (Figure 5A, 5B). In doing so, the native articular geometry is restored, accounting for the patient-specific cartilage thickness, without having to assume a thickness of 2 mm. Once this is set for the unworn side, the planned bony resection on the worn side of the femur is adjusted to match the planned bony resection on the unworn side of the femur, both distally and posteriorly. Rotation is then checked to ensure the trochlea of the femoral component and native femur are aligned (Figure 6). If they are not aligned, the surgeon may choose the optimal compromise between recreating femoral rotation and trochlear anatomy. With this, the femoral component position is finalized and left untouched for the remainder of the case.

**Figure 7:**
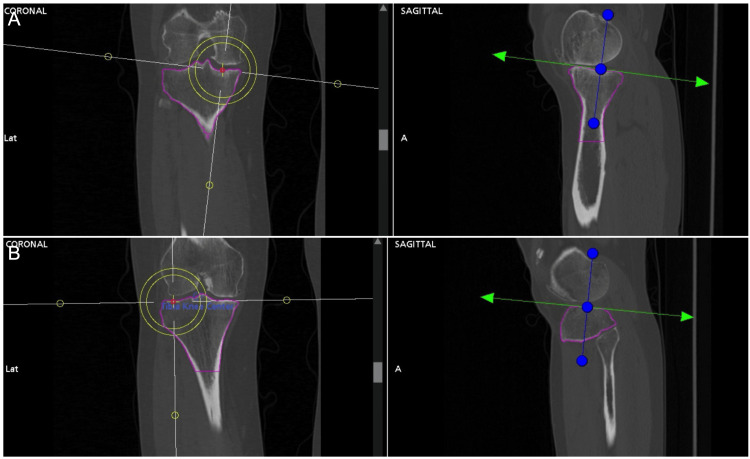
Defining the tibial slope (A) The medial tibial slope measured in the sagittal plane (green line) is estimated at an articulation point with the medial femoral condyle in the coronal plane. (B) The lateral tibial slope (green line) measured in the sagittal plane is estimated at an articulation point with the lateral femoral condyle in the coronal plane.

The posterior slope of the tibial cut is set to match the patient’s anatomic slope. Since the slopes of the medial and lateral tibia can be discordant, we typically average the two slopes, which is calculated by the robotic software preoperatively. Specifically, tibial slope is calculated by a line drawn in the sagittal plane that best represents the native slope (accounting for bone loss if present) at a point in the coronal plane where the femur articulates with the tibia (Figure 7A, 7B). The knee is then “balanced” by adjusting the tibial cut - both in terms of depth of resection and varus/valgus angle. Adjustments are made to achieve a rectangular extension gap with minimal varus/valgus laxity and a trapezoidal flexion gap with variable lateral laxity, in an attempt to recreate physiologic soft tissue tension. However, depending on surgeon preference, equal medial and lateral tibial resections (accounting for estimated potential tibial bone loss) may be programmed in accordance with the strict KA technique.

Executing the cuts

The robotic arm is brought into the surgical field, and five geometric femoral cuts and a tibial cut are then performed, ensuring careful protection of the soft tissues.

After completing the cuts, the robot is removed from the surgical field. Bone cuts are removed, the menisci are excised, and any residual posterior osteophytes are eliminated.

Trialing

**Figure 8:**
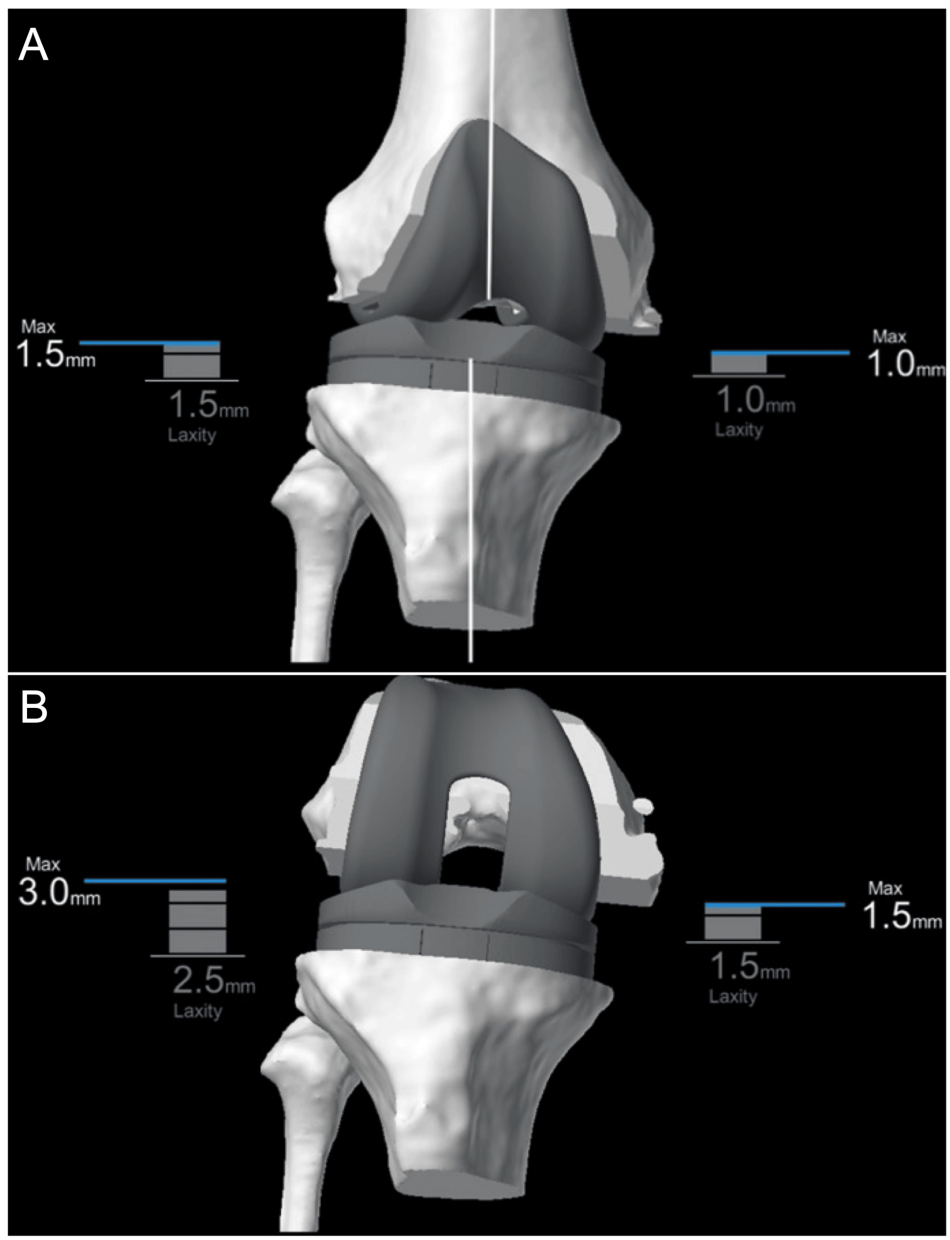
Evaluating extension and flexion gaps with trial components (A) The extension gap demonstrates a rectangular shape (1.0 mm medial gap and 1.5 mm lateral gap), while (B) the flexion gap demonstrates a trapezoidal shape (1.5 mm medial gap and 3.0 mm lateral gap). This resembles the physiologic knee with increased lateral laxity.

**Figure 9:**
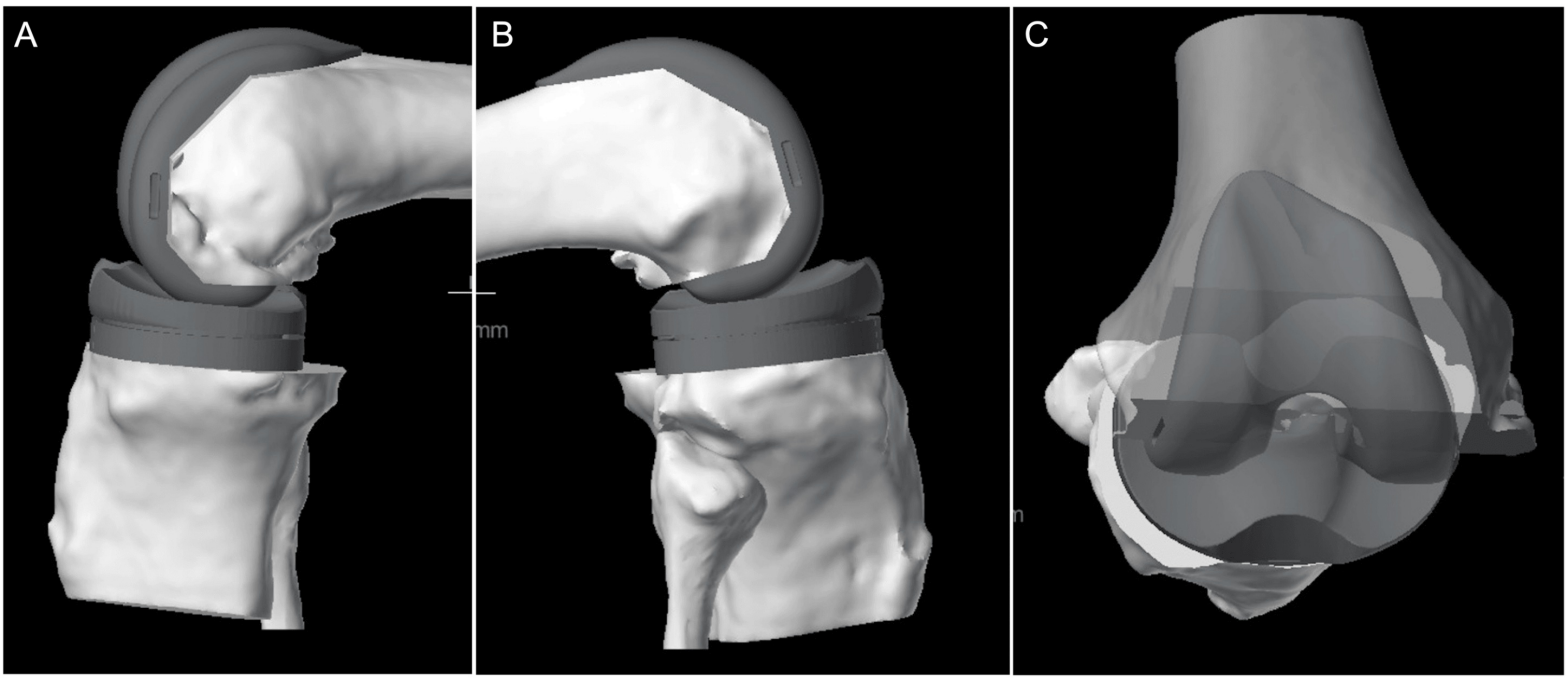
Evaluating knee kinematics (A) A medial view of the knee in ~100 degrees of flexion demonstrating a relatively central contact point of the femur on the tibia. (B) A lateral view of the knee in ~100 degrees of flexion demonstrating posterior rollback of the lateral femoral condyle on the tibial plateau. (C) A “bird’s eye” view of the knee in deep flexion (~120 degrees) showing lateral femoral rollback, a more posterior contact point of the lateral femur relative to the medial femur.

Trial components are placed. The knee is then examined, evaluating the extension gap, flexion gap, range of motion, and patellar tracking. We aim for a rectangular extension gap and a trapezoidal flexion gap with lateral laxity to closely resemble the physiologic knee (Figure 8A, 8B). The kinematics of the knee are also evaluated from a medial, lateral, and “bird’s eye” simulated robotic view (Figure 9A-9C). Soft tissue releases and bony recuts are rarely, if ever, performed. The tibial component is “floated”, allowing knee kinematics to define tibial component rotation. Adequate patellar tracking is verified. The femoral lug holes are drilled, and tibial rotation is marked. The trial implants are removed, and the tibia is prepared to accept the final implant.

Final implant placement

**Figure 10:**
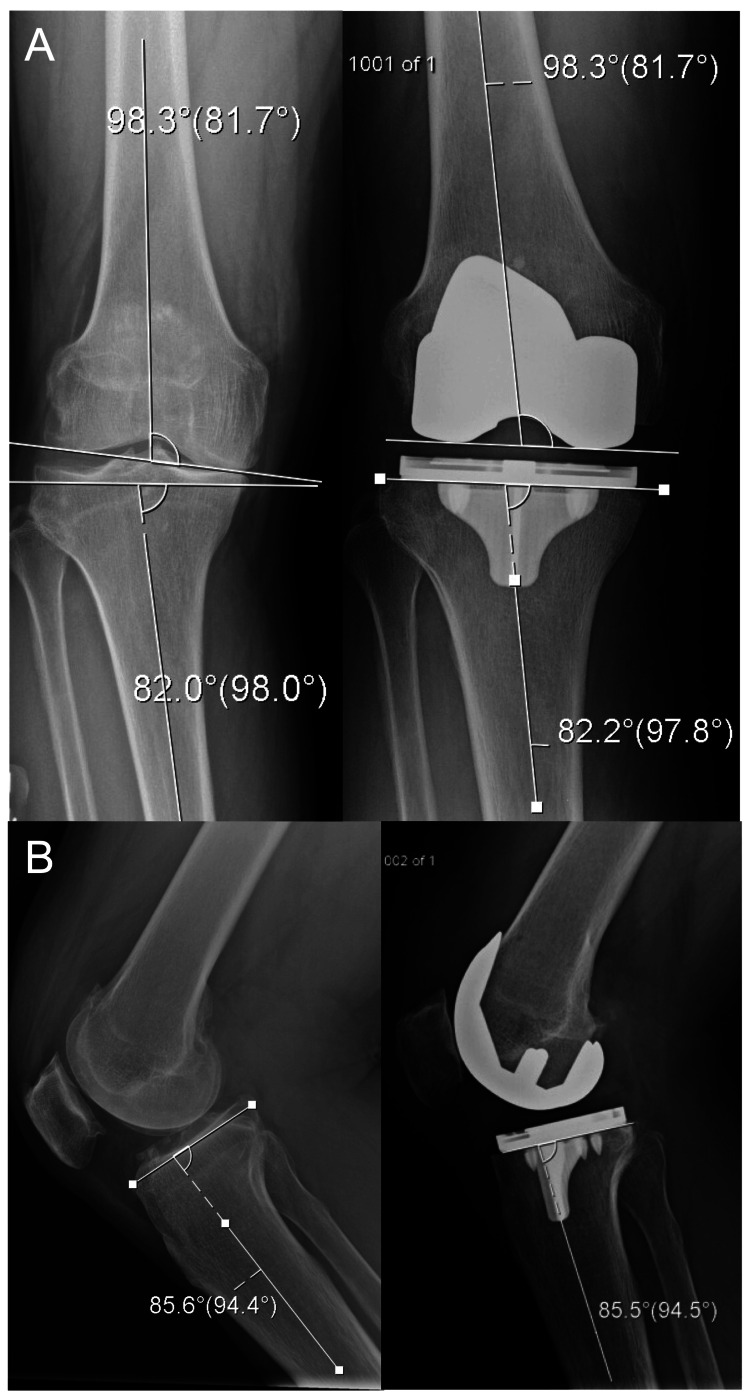
Preoperative and postoperative radiographs (A) AP and (B) lateral radiographs demonstrating a robotically assisted kinematically aligned TKA.

The tibial component is press-fit or cemented into place, depending on bone quality, followed by the femoral component. The final polyethylene liner is impacted into place. Closure is performed in standard fashion. Preoperative and postoperative radiographs are shown in Figure 10.

Common pitfalls

There are a few common pitfalls of this technique that are critical to avoid.

Choosing Inaccurate Limb Axes Points or Bony Resection Landmarks

Typically, this step is performed by the robotic product specialist without surgeon guidance. In a functional alignment plan, errors in bony landmark placement are not penalized since the overall goal of functional alignment (moving components in three-dimensional space, within bounds, to achieve a desired soft tissue balance) can still be accomplished. In KA, the goal is to match a specific pre-defined alignment target, so millimeters of inaccuracy matter. Therefore, we recommend that the surgeon and robotic product specialist choose these points together for the first ~20 cases to ensure accuracy, consistency, and understanding.

Inadequate Exposure of the Unworn Femoral Condyle

Since adequate exposure of the unworn femoral condyle both posteriorly and distally is necessary for intraoperative cartilage mapping, we generally make a slightly larger incision for these cases than for other non-robotic-assisted KA cases. Removing the anterior horn of the meniscus on the unworn side of the knee can help facilitate exposure for posterior condyle cartilage mapping.

Underestimation of the Tibial Slope

We have found that the robotic platform underestimates the true tibial slope by approximately 1-2 degrees, which has been corroborated in the literature [30,31]. We account for this in our surgeries. Specifically, when the desired slope target is 5 degrees, a 6-7 degree slope is planned on the robotic software.

The CT Protocol Is Based on the Transepicondylar Axis, Not the Transcondylar Axis

In the most current version of the robotic software, the CT protocol is designed for MA; components can only be positioned in the sagittal plane relative to the transepicondylar axis, which is an irrelevant axis in KA philosophy. Changing the CT gantry to parallel the transcondylar axis, the true flexion/extension axis of the knee, is not currently allowed by the robotic software.

## Discussion

TKA has evolved substantially over the past several decades, with modern implants demonstrating excellent long-term survivorship [5]. However, patient dissatisfaction in the absence of technical deficiencies remains a challenge, with 10-20% of patients reporting persistent pain, functional limitations, or unmet preoperative expectations [6,32,33]. This recognition has prompted growing interest in alternative alignment strategies aimed at improving patient-reported outcomes. KA represents one such approach, which attempts to replicate a patient’s pre-arthritic joint line, limb alignment, and soft-tissue balance by resurfacing the distal femur and proximal tibia [4,15,17,18].

The technique highlighted in this article describes a practical and efficient workflow that integrates KA principles with the precision of robotic technology. Intraoperative cartilage mapping enables patient-specific replication of cartilaginous knee geometry, aiding in restoration of native joint line height and obliquity. Disadvantages of this technique include a potential learning curve with increased operative time, increased cost of the robot, the inability to switch implant vendors, and possible pin site complications such as fracture or infection [34-36]. Table 1 and Table 2 summarize the results of randomized controlled trials over the past decade comparing robotic and manual TKA, as well as KA and MA strategies, respectively [7,20,22,37-52].

**Table 1:** Summary of randomized controlled trials comparing robotic-assisted and manual total knee arthroplasty over the past decade RA, robotic-assisted; MA, mechanical alignment; FA, functional alignment; MCID, minimal clinically important difference; HKA, hip-knee-ankle angle; WOMAC, Western Ontario and McMaster Universities Osteoarthritis Index; KOOS, Knee Injury and Osteoarthritis Outcome Score; OKS, Oxford Knee Score; KSS, Knee Society Score; VAS, visual analogue scale; UCLA, University of California Los Angeles; SF-36, Short Form-36; ROM, range of motion; min, minutes *denotes alignment strategy not indicated.

Study	Intervention (# of patients)	Follow-up	Key findings
Geng et al., 2024 [37]	RA* (63) vs. manual MA (62)	6 weeks	Alignment: RA had greater inliers (HKA <3°) (78.7% vs. 51.6%, p < 0.05). PROMs/Function: No difference in KSS and WOMAC scores. Operative time: RA 119.5 min vs. manual 85.0 min (p < 0.01). Complications: No difference.
Adamska et al., 2023 [38]	Imageless RA-MA (76/71) vs. manual MA (68)	1 years	Alignment: RA achieved more accurate femoral component rotational alignment (p = 0.0013). PROMs/function: RA had higher KOOS scores (p = 0.0001), although this did not meet MCID. No difference in VAS score and range of motion. Complications: No difference.
Bollars et al., 2023 [39]	RA-FA (26) vs. manual MA (26)	6 weeks	Alignment: RA had fewer overall outliers (>3°) (5.8% vs. 24.4%, p < 0.001). Femoral component position, HKA, and tibial slope were more accurate with RA (p < 0.05).
Tian et al., 2023 [40]	RA* (72) vs. manual MA (72)	12 weeks	Alignment: RA had fewer outliers (HKA >3°) (3.2% vs. 41.0%, p < 0.001). PROMs/function: No difference in KSS or VAS scores or knee ROM. Operative time: RA 155.7 min vs. manual 107.5 min (p < 0.001). Complications: No difference.
Li et al., 2022 [7]	RA* (73) vs. manual MA (77)	3 months	Alignment: RA had fewer outliers (HKA >3°) (18.8% vs. 36.5%, p < 0.02). PROMs/function: No difference in knee flexion, WOMAC/HSS/SF-36/KSS scores.
Thiengwittayaporn et al., 2021 [41]	Imageless RA* (75) vs. manual MA (77)	6 weeks	Alignment: RA had fewer outliers (HKA >3°) (5.3% vs. 15.6%, p = 0.035). RA had better accuracy of knee alignment and component positioning (mechanical axis (RA 178.4° vs. manual 177.9°, p = 0.009), femoral inclination in the sagittal plane (RA 1.9° vs. manual 5.4°, p < 0.001), and tibial inclination in the coronal plane (RA 88.5° vs. manual 87.9°, p = 0.012) and sagittal plane (RA 86.0° vs. manual 85.1°, p = 0.035). RA had fewer changes in the joint line (3.6 mm vs. 5.5 mm, p = 0.004) and posterior femoral offset (4.4 mm vs. 6.5 mm, p = 0.001). Operative time: RA 70.1 min vs. manual 61.9 min (p < 0.001).
Vaidya et al., 2020 [42]	Imageless RA-MA (32) vs. manual MA (28)	n/a	Alignment: RA had fewer outliers (HKA >3°) (3.1% vs. 28.5%, p = 0.01). RA had less joint line deviation (0.9 mm vs. 3.5 mm, p < 0.001).
Kim et al., 2019 [43]	RA* (700) vs. manual MA (706)	10-15 years	Alignment: No difference in coronal/sagittal component position or limb alignment. PROMs/Function: No difference in KSS, WOMAC, and UCLA scores or knee ROM. Survivorship: Aseptic loosening 2% each; 98% survivorship in both groups (p = 0.972). Operative time: RA 97 min vs. manual 69 min (p < 0.001). Complications: No difference.
Liow et al., 2017 [44]	RA-MA (31) vs. manual MA (29)	2 years	PROMs/Function: No difference in knee ROM, OKS, and KSS scores. No difference in the proportion of patients who achieved MCID in OKS and KSS. RA had higher scores in SF-36 vitality (p = 0.03), role emotional (p = 0.02), and a larger proportion of patients achieving SF-36 vitality MCID (48.4% vs. 13.8 %, p = 0.009). Complications: No differences.

**Table 2:** Summary of randomized controlled trials comparing kinematic and mechanical alignment in total knee arthroplasty over the past decade RA, robotic-assisted; KA, kinematic alignment; MA, mechanical alignment; PSI, patient-specific instrumentation; HKA, hip-knee-ankle angle; LDFA, lateral distal femoral angle; MPTA, medial proximal tibial angle; HSS, Hospital for Special Surgery; WOMAC, Western Ontario and McMaster Universities Osteoarthritis Index; KOOS, Knee Injury and Osteoarthritis Outcome Score; OKS, Oxford Knee Score; FJS, Forgotten Joint Score; KSS, Knee Society Score; VAS, visual analogue scale; UCLA, University of California Los Angeles; SF-36, Short Form-36; EQ-5D, Euro-Qol; ROM, range of motion; MD, mean difference; min, minutes

Study	Intervention (# of patients)	Follow-up	Key findings
Sarzaeem et al., 2024 [45]	Manual caliper KA (65) vs. manual MA (65) (bilateral simultaneous surgery)	2 years	Alignment: No difference in HKA, LDFA, or MPTA. PROMs/Function: KA had faster recovery time (6.7 vs. 10 weeks, p < 0.001), better WOMAC (23.1 vs. 33.3, p = 0.01) and FJS (80.2 vs. 76.6, p = 0.02) scores, but no difference in OKS and VAS scores. KA had greater maximum knee flexion (139.1 vs. 131.2, p < 0.001). Operative time: KA 43.2 min vs. MA 52.1 min (p < 0.001). Complications: No prosthetic failure or revision in either group.
Clement et al., 2024 [46]	Robotic KA (43) vs. manual MA (38)	1 year	PROMs/Function: KA had a clinically meaningful improvement in WOMAC pain reduction (MD 9.7, p = 0.029). No difference in knee-specific measures (WOMAC, OKS, FJS) or health-related quality of life measures (EQ-5D and EQ-VAS) overall. Complications: No difference.
Wang et al., 2024 [47]	Manual PSI-KA (19) vs. manual MA (18)	2 years	Alignment: Postoperative HKA (KA 176° vs. MA 178°, p = 0.01) and MPTA (KA 86° vs. MA 90°, p < 0.001). PROMs/Function: KA had higher OKS scores at one year (KA 41.37 vs. MA 37.02, p = 0.02); however, no difference at two years (KA 42.37 vs. MA 41.34, p = 0.42). Pedobarographic analysis: MA had greater medial pressure distribution in the forefoot compared to KA (p < 0.001) and the contralateral native knee (p = 0.002). MA had greater lateral pressure distribution in the rearfoot compared to KA (p = 0.007) and the contralateral native knee (p = 0.001). There was no difference in medial/lateral pressure distribution in the forefoot and rearfoot between the KA and native groups (p = 0.064 and p = 0.802, respectively).
Dossett et al., 2023 [20]	Manual PSI-KA (44) vs. manual MA (44)	13 years	PROMs/Function: KA trended towards higher satisfaction (96% vs. 82%, p = 0.16). Otherwise, no difference in functional scores (WOMAC, OKS, KOOS, FJS). Complications: No difference in major/minor reoperations.
MacDessi et al., 2020 [48]	CN-KA (63) vs. CN-MA (62)	1 year	Alignment: KA had greater LDFA valgus angulation (KA 89.2° vs. MA 90.6°, p < 0.001). KA had greater MPTA varus angulation (KA 88.9° vs. MA 90.0°, p = 0.003). No difference in HKA. PROMs/Function: No difference in KOOS, FJS-12, or EQ-5D-5L scores. Pressure analysis: MA had greater intercompartmental pressure at 10° (MD 20.3, p < 0.001), 45° (MD 10.3, p = 0.004), and 90° (MD 7.4, p = 0.002) knee flexion. Balance analysis: KA was more likely to achieve optimal knee balance (80% vs. 35%, p < 0.001). Intraoperative: MA required more additional bony resections (MA 49% vs. KA 9%, p < 0.001) and soft tissue releases (22% vs. 7%, p = 0.013) to achieve optimal knee balance. Operative time: No difference.
McEwen et al., 2020 [49]	CN-KA (41) vs. CN-MA (41) (bilateral simultaneous surgery)	2 years	Alignment: No difference in standing HKA. PROMs/Function: No difference in KOOS, KOOS JR, OKS, and FJS scores and knee ROM. More patients favored KA over MA (p = 0.03); however, half of the patients had no preference. Intraoperative: MA required more soft tissue releases (p = 0.018).
Laende et al., 2019 [50]	Manual PSI-KA (24) vs. CN-MA (23)	2 years	Alignment: No difference in longitudinal tibial component migration (KA 0.40 mm vs. MA 0.37 mm, p = 0.82). No association between postoperative varus alignment and tibial component migration (r = -0.02, p = 0.50). PROMs/Function: No difference in OKS, VAS satisfaction, and UCLA activity scores.
Yeo et al., 2019 [51]	RA-KA (30) vs. RA-MA (30)	8 years	Alignment: No difference in the mechanical axis and sagittal inclination of the femoral and tibial components. PROMs/Functional: No difference in HSS, WOMAC, ROM, KSS pain or function score. No difference in varus/valgus laxity assessment. No difference in gait analysis. Complications: No difference.
Waterson et al., 2016 [22]	Manual PSI-KA (36) vs. manual MA (35)	1 year	Alignment: 78% of KA and 77% of MA knees were within 3° of their preoperative plan. PROMs/Function: KA had greater improvement in KSS at six weeks (p = 0.05), but no difference at one year. KA had a slight improvement in SF-36 (physical) at six months (p = 0.04), but no difference at one year. No difference in KOOS, UCLA, and EQ-5D scores and knee ROM at any time point. KA had better peak torque in quadriceps at six weeks and three months (p = 0.003 and p = 0.02, respectively), but no difference at one year.
Calliess et al., 2016 [52]	Manual PSI-KA (100) vs. manual MA (100)	1 year	Alignment: MA achieved a postoperative limb alignment of 1° varus, while KA achieved an alignment of 1° valgus. PROMs/Function: KA had greater improvement in KSS and WOMAC scores (p = 0.02 and p = 0.001, respectively). In the KA group, there was a correlation between deviation of the postoperative alignment from the initial preoperative KA plan and poor KSS score (r = −0.22; p = 0.02). In the KA group, there was a correlation between a high flexed femoral component and a high WOMAC score (r = 0.34; p = 0.005). Complications: No difference.

Robotic-assisted kinematically aligned TKA has potential future benefits. The capacity to capture and analyze intraoperative data, including cartilage thickness, patellofemoral offset, trochlear angles, and femoral flexion, may inform artificial intelligence-driven predictive models to better understand patient satisfaction, survivorship, and implant design. Additionally, this technique holds promise in potentially identifying subsets of patients less likely to benefit from the KA technique, such as those with irreversibly attenuated collateral ligaments, severe bone loss, or substantial preoperative deformity.

## Conclusions

Robotic-assisted kinematically aligned TKA aims to restore the patient’s pre-arthritic joint line, limb alignment, and soft tissue balance by leveraging a CT-based robotic platform to map cartilage intraoperatively and guide patient-specific component placement. Unlike manual caliper-guided KA technique, our approach does not rely on cartilage thickness assumptions. Theoretical advantages include enhanced accuracy, reproducibility, and restoration of native knee kinematics, while potential disadvantages include higher cost, longer operative time, a larger incision, and a learning curve. This technical report provides surgeons with an efficient workflow to execute a kinematically aligned TKA with robotic precision. Future research will be needed to determine the long-term survivorship and clinical outcomes relative to other alignment strategies.
